# Adult attention deficit hyperactivity disorder in Australia: how its current commercial model for diagnosis and treatment is encouraging misdiagnosis

**DOI:** 10.5694/mja2.70049

**Published:** 2025-09-10

**Authors:** Richard CJ Bradlow, Ferghal Armstrong, Edward Ogden

**Affiliations:** ^1^ Austin Health Melbourne VIC; ^2^ Turning Point Alcohol and Drug Centre Melbourne VIC; ^3^ Swinburne University of Technology Melbourne VIC

**Keywords:** Attention deficit disorder with hyperactivity, Legislation, medical

Attention deficit hyperactivity disorder (ADHD) in adults is a significant public health issue. It can be associated with adverse outcomes such as educational underachievement, reduced productivity, substance use disorders, involvement in crime, and increased morbidity and mortality.^1^

From 2013 to 2020, the number of Australians diagnosed with ADHD more than doubled,^2^ and by 2022–2023, about 470 000 individuals were prescribed ADHD medications — an increase of approximately 300% in ten years.^3^ This rise, particularly pronounced among adults, is attributed to growing public awareness,^4^ amplified by social media platforms such as TikTok where ADHD‐related content is reported to have had over 36 billion views.^3^

Most adults with ADHD are diagnosed by private psychiatrists. The dearth of public services for ADHD raises serious concerns regarding equity of access and the potential that normal behavioural variability is “medicalised”.

The growing prevalence of ADHD diagnoses and stimulant prescriptions is controversial, with concerns that many prescribers may not be adhering to relevant prescribing codes.^5^ There is a risk that complex psychosocial issues may be misattributed to ADHD.^6^ This latter concern is grounded in psychiatry’s history of over‐simplified biological explanations to complex psychosocial causes.^7^

The greater availability of stimulants in the community has contributed to misuse and diversion, particularly in adolescents and young adults for study or recreational activities. In the 2022–2023 national drug strategy household survey, 2.1% of adults report using prescribed stimulants for non‐medical purposes in the past year. The highest usage was in the 20–29 year age group where 4.8% reported non‐prescribed use in the past year.^8^ Although the rise in stimulant prescriptions has not led to an increase in stimulant‐related deaths,^9^ there has been a rise in hospital presentations for stimulant‐related poisonings.^10^

## Significance of functional impairment

The private model of ADHD diagnosis and treatment in Australia means that patients are often obliged to pay thousands of dollars and spend time on waiting lists before they can be assessed.^11^ This system selects out the people who have the financial capacity to afford the high medical costs and who possess the patience and organisational skills to navigate the complicated system, possibly excluding people who do not have the means to access private psychiatry. ADHD Foundation Australia notes that obtaining an appointment with a psychiatrist is “extremely difficult” and the situation for obtaining diagnosis and treatment is described as having reached a “crisis point”.^12^ Complex psychosocial issues, such as anxiety, depression or trauma, may be misattributed to ADHD without adequate exploration of underlying causes.^7^ This is more likely in adults than in children as paediatric assessments often include collateral information from parents and teachers. Social media‐driven self‐diagnosis, often based on simplistic online tests, exacerbates this issue, as these tests lack the rigour of comprehensive assessments.^11^ The absence of objective diagnostic markers increases the risk of misdiagnosis.

A key diagnostic criterion for ADHD is evidence of impaired functioning.^13^ Given the controversy around the increased prevalence and treatment of ADHD, the criterion of “impaired functioning” takes on greater salience. There are no standardised definitions of “functional impairment” nor mechanisms to assess the compensatory strategies that may mask symptoms (eg, support from partners, coaching). This ambiguity facilitates diagnostic variability and potential overdiagnosis.

Accurate diagnosis and effective treatment of ADHD can be transformative for individuals and their families. Treating adult ADHD is associated with substantial improvements in multiple domains of social and psychological functioning.^14^ Appropriate interventions reduce core symptoms of inattention, impulsivity, and hyperactivity, leading to better educational and occupational performance, improved interpersonal relationships, and an enhanced quality of life.^14^

The benefits of treating adult ADHD are well established across clinical, occupational, and psychosocial domains. A comprehensive meta‐analysis of 113 randomised controlled trials involving over 14 800 adults confirmed that stimulant medications (such as methylphenidate and lisdexamfetamine) and the non‐stimulant atomoxetine are effective in reducing core ADHD symptoms, with good acceptability and safety profiles.^15^ When left untreated, ADHD is associated with poor educational outcomes, unemployment, increased risk of substance use disorders, and a higher likelihood of criminal offending and incarceration.^16^, ^17^, ^18^

Paradoxically, the impairments caused by untreated ADHD — particularly financial instability and poor executive functioning — make it more difficult for affected individuals to navigate the complex and costly process required to obtain a formal diagnosis.

A profit‐driven, exclusively private diagnostic model could favour individuals with financial means and well developed organisational skills — traits often seen in higher‐functioning individuals who may be at risk of overdiagnosis. Conversely, those whose functioning is impaired by untreated ADHD may be less likely to access assessment and treatment in this system.

The current system therefore risks overdiagnosing ADHD in individuals whose relatively intact functioning enables them to navigate the diagnostic process, while simultaneously failing those whose impairments are so severe that they are unable to access assessment at all.

## High fees affecting diagnosis

Given that the assessment of ADHD involves procedures no more complex or time consuming than assessment of other more complex mental health conditions, it is unclear why ADHD evaluations should be more expensive. Psychiatrists charging elevated fees for ADHD assessments could unintentionally create a situation in which the patients expect the diagnosis and the psychiatrists feel pressured to give the diagnosis.^11^ The proliferation of single‐session online ADHD clinics, with very limited follow‐up provided poses additional ethical concerns.

## Solutions

Addressing these challenges requires systemic reform, including a greater emphasis on functional impairment as a diagnostic criterion. The *Australian evidence‐based clinical practice guideline for ADHD*, published in 2022, provides evidence‐based recommendations for diagnosis and treatment, emphasising comprehensive assessments.^14^

ADHD has a higher prevalence in the psychiatric population than the general adult population.^19^ One literature review found prevalence rates ranging from 6.9% to 38.75%.^20^ Yet public mental health services tend to ignore the reality and rarely provide treatment. Public health has a critical role in addressing the challenge of training young psychiatrists and providing high quality care to their patients.

To improve access, public health should develop dedicated ADHD clinics within existing mental health services. This would allow training and credentialling of generalist clinicians in standardised ADHD diagnostic protocols to broaden service capacity and reduce reliance on expensive private sector assessments. For patients with more complex mental health comorbidities, multidisciplinary assessment and treatment pathways should be developed.

Public health should prioritise outreach to marginalised groups, so that ADHD assessment and treatment could be integrated into services for individuals with comorbid substance use, justice system involvement, or other social disadvantages.

Early intervention initiatives to diagnose ADHD in childhood, in collaboration with the education and primary care sectors, would enable earlier identification of functional impairment, reduce the burden of untreated ADHD, improve educational outcomes and help prevent long term consequences such as substance misuse and incarceration.

Although access has been increased to individuals who need it through the public system, greater oversight of the practices of private clinics needs to occur to reduce overdiagnosis. This could include auditing of the diagnostic practices, and appropriate precautions taken in prescribing.

Several Australian jurisdictions have recently announced moves towards allowing general practitioners to diagnose and treat ADHD.^21^ These announcements foreshadow specific training for practitioners in recognition of ADHD and comorbidities. The Australasian ADHD Professionals Association has developed the *Australian evidence‐based clinical practice guideline for ADHD*, which provides the basis for consistent training and clinical decision making. Implementation of these guidelines in community practice has the potential to improve access to timely diagnosis and management, particularly for adults and those in regional or underserved areas, provided that training is comprehensive and supported by appropriate referral pathways and oversight.^14^ Without adequate training and oversight, this general practitioner‐led solution could be at risk of increasing overdiagnosis in people without functional impairment.

Australia must move towards a more ethical, evidence‐based, and equitable system of ADHD care. The cost of adult ADHD to the community is related to underperformance and failure in education, difficulties at work, involvement in crime and/or development of substance use disorders. In 2019, ADHD was estimated to cost the Australian community $20 billion per year.^17^ Excellent treatment of ADHD makes good economic and social sense.

## Open access

Open access publishing facilitated by Monash University, as part of the Wiley – Monash University agreement via the Council of Australian University Librarians.

## Competing interests

Ferghal Armstrong has received honoraria for public speaking and consultancy work from Indivior, AbbVie and Seqirus.

## Provenance

Not commissioned; externally peer reviewed.

## Author contributions

Bradlow RCJ: Conceptualization, writing – original draft, writing – review and editing. Armstrong F: Conceptualization, writing – review and editing. Ogden E: Conceptualization, writing – review and editing.
